# Signatures of human impact on self-organized vegetation in the Horn of Africa

**DOI:** 10.1038/s41598-018-22075-5

**Published:** 2018-02-26

**Authors:** Karna Gowda, Sarah Iams, Mary Silber

**Affiliations:** 1 Department of Engineering Sciences and Applied Mathematics, Northwestern University, Evanston, IL 60208 USA; 2 Paulson School of Engineering and Applied Sciences, Harvard University, Cambridge, MA 02138 USA; 3 Committee on Computational and Applied Mathematics, and Department of Statistics, University of Chicago, Chicago, IL 60637 USA

## Abstract

In many dryland environments, vegetation self-organizes into bands that can be clearly identified in remotely-sensed imagery. The status of individual bands can be tracked over time, allowing for a detailed remote analysis of how human populations affect the vital balance of dryland ecosystems. In this study, we characterize vegetation change in areas of the Horn of Africa where imagery taken in the early 1950s is available. We find that substantial change is associated with steep increases in human activity, which we infer primarily through the extent of road and dirt track development. A seemingly paradoxical signature of human impact appears as an increase in the widths of the vegetation bands, which effectively increases the extent of vegetation cover in many areas. We show that this widening occurs due to altered rates of vegetation colonization and mortality at the edges of the bands, and conjecture that such changes are driven by human-induced shifts in plant species composition. Our findings suggest signatures of human impact that may aid in identifying and monitoring vulnerable drylands in the Horn of Africa.

## Introduction

Bands of vegetation separated by stretches of bare ground on gradually-sloping terrain are widespread in the drylands of Africa, North America, and Australia^1,2^. Vegetation bands often occur on pastoral lands^3,4^, and serve as crucial buffers against erosion^5,6^. In some highly arid parts of the Horn of Africa (e.g., the Sool Plateau of Somalia), bands comprise the bulk of vegetation on the landscape. In general, dryland vegetation is susceptible to degradation due to overgrazing and changes in land use by human populations^7,8^, both of which are relevant factors in the Horn of Africa^3^. Vegetation bands in this region are placed at additional risk by a recent multi-decadal decline in rainfall during the long rainy season (March–May)^9^, since large rainfall events during this season generate surface water runoff that is important for the maintenance of the bands^10^. Vegetation bands are important hotspots of productivity in the changing and understudied drylands of the Horn of Africa^11,12^, and understanding their response to human and climatic pressures can inform future conservation and relief efforts.

Mathematical models account for the emergence of vegetation bands via a self-organizing interaction between vegetation and water resources^13–16^. Investigations of these models have sought signals of imminent catastrophic vegetation collapse in response to environmental change^17,18^. Most notably, the spacing between bands is predicted to increase in response to increases in aridity^19–23^ and local disturbances (e.g., grazing pressure)^24^, resulting in overall reduced vegetation cover. In principle, reduced vegetation cover can also result from decreases in band width (the span of vegetation cover measured in the direction of local slope), but relatively little theoretical work has focused on this property of the bands. Previous empirical studies in Niger report reduced vegetation cover via decreased band width during multi-year periods of low rainfall^5,25^. Reductions in cover in both studies were also associated with increased human activity, underscoring the importance of human impacts on vegetation change.

**Figure 1:**
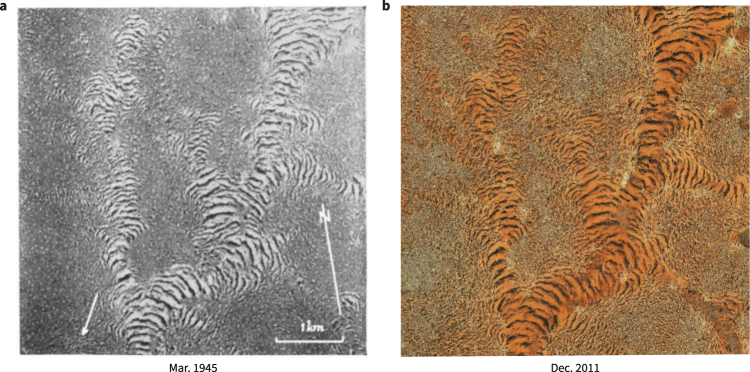
Aerial survey photographs taken over the Horn of Africa in the 1940s and 50s can be precisely referenced against modern imagery, enabling tracking of individual bands over a long period of time. (**a**) Aerial photograph adapted from plate 10 in^26^, with an arrow indicating the downslope direction. (**b**) Modern satellite image aligned to the photograph in (**a**) (7.85°N, 47.41°E). Images Royal Geographical Society, Google, DigitalGlobe.

**Figure 2:**
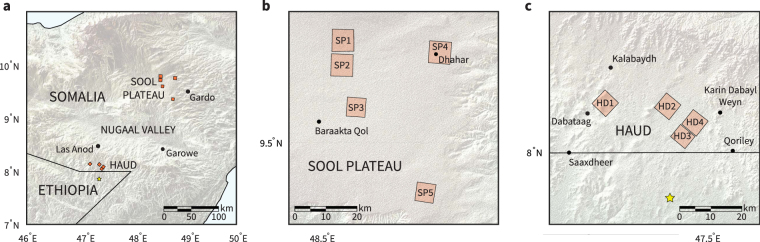
Areas studied in this investigation are defined by nine distinct aerial survey photographs taken in 1952. (**a**) Study areas are clustered in two regions of Somalia separated by the Nugaal Valley. (**b**) SP1–SP5 are located in the Sool Plateau pastoral region of Somalia. (**c**) HD1–HD4 are located in the Haud pastoral region of Somalia. Location of the example images shown in Fig. 1 is indicated with a star. Each photograph covers an approximate area of 50 km^2^. Relief maps rendered using the Shuttle Radar Topography Mission Global 1 arc second elevation dataset^41^ in MATLAB 2016b.

Aerial survey photographs dating back to the 1940s and 50s were crucial to the initial discovery and characterization of vegetation bands in the Horn of Africa^26,27^ (Fig. 1). In the present study, we georeferenced high-resolution scans of survey photographs taken in 1952 and reconnaissance satellite imagery taken in 1967 against modern imagery to investigate the multidecadal dynamics of nearly 3,500 distinct vegetation bands. Our study areas cover approximately 450 km^2^ of the Sool Plateau and Haud pastoral regions of Somalia (Fig. 2). Among these areas are locations that remain relatively unchanged, and also locations that have experienced a dramatic increase in human activity. All study areas experienced multi-year fluctuations in rainfall and a warming trend over the last half-century (Supplementary Information). We employed a systematic visual comparison of imagery to assess the extent of human activity and vegetation degradation, as well as automated transect-based measurements and comparative Fourier analysis to quantify key aspects of vegetation change.

## Results

### Human activity and vegetation degradation

**Figure 3:**
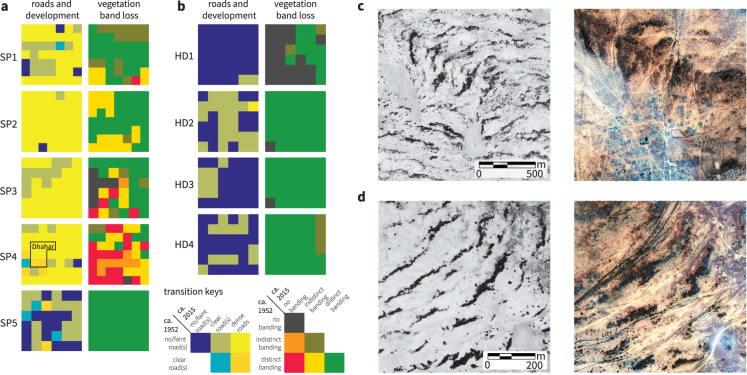
Vegetation loss occurs in areas with the steepest increases in human activity. The qualitative state of road and track cover, settlements, and vegetation banding was assessed visually in 1 km^2^ boxes. Transitions between states observed in aerial photography and recent satellite imagery are shown here for all study areas. (**a**) SP1–SP4 in the Sool Plateau show a high degree of road and track development, and a moderate to high degree of band loss. A large settlement (Dhahar) developed within SP4, and is indicated with a black border. SP5 saw little increase in road cover, and no band loss or degradation was observed. (**b**) Areas in the Haud (HD1–HD4) show only a small increase of road and track development, and no substantial band loss or degradation is observed. (**c**) An example of band loss and degradation due to land development in SP4 (9.76°N, 48.82°E; left: 02/22/1952, grayscale; right: 08/16/2016, RGB). (**d**) An example of band degradation amid dense track cover in SP3 (9.58°N, 48.57°E; left: 11/29/1952, grayscale; right: 12/03/2011, RGB). Images courtesy of the Bodleian Library and the DigitalGlobe Foundation.

We assessed changes in human activity and vegetation banding over time through a systematic visual comparison of British Royal Air Force (R.A.F.) aerial survey photography taken in 1952 and recent satellite imagery (Methods, Supplementary Information). We found that band degradation ranged from partial to complete band loss in areas with the steepest increases in human activity, while bands in the other areas remain largely unchanged (Fig. 3). Roads and dirt tracks can be visually identified in both the aerial photographs and the satellite imagery, and their presence and qualitative appearance served as our primary proxy for inferring the extent of human activity (Supplementary Figure S4). We defined degradation in this study as either the breakdown in regularity or the disappearance of banding (Supplementary Figure S5).

Substantial road and track development occurred in much of the Sool Plateau, with most areas (SP1–SP4) transitioning from having either no roads or faint roads in 1952 to having roads or tracks that densely cover the landscape in the modern images (Fig. 3a). The settlement Dhahar was founded within SP4 after the 1952 photograph, and now supports a population of approximately 13,000 (Fig. 3c). We observed much less road and track development in Sool Plateau area SP5 and in all Haud areas (HD1–HD4) (Fig. 3b). At many sites within the Haud, human-made structures visible in the 1952 images seem to persist into the current decade, suggesting no major change in land use over the intervening time (Supplementary Figure S6a).

Vegetation degradation is prevalent in the human-impacted areas SP1–SP4 (Fig. 3a). Bands have disappeared entirely from the landscape in large parts of SP3 and SP4. Only part of the band loss in these areas seems directly related to clearing for land development, since loss also occurs in areas without human-made structures. In SP1–SP4, dense tracks often appear between bands (Fig. 3d). Frequently we observed vegetation growing within roads and tracks, which suggests that these structures likely disrupt the flow of water on the landscape. In SP5 and HD1–HD4, individual bands often remain identifiable after six decades based on visible details of their morphology, and we observed no substantial band degradation.

### Band widening in human-impacted areas

**Figure 4:**
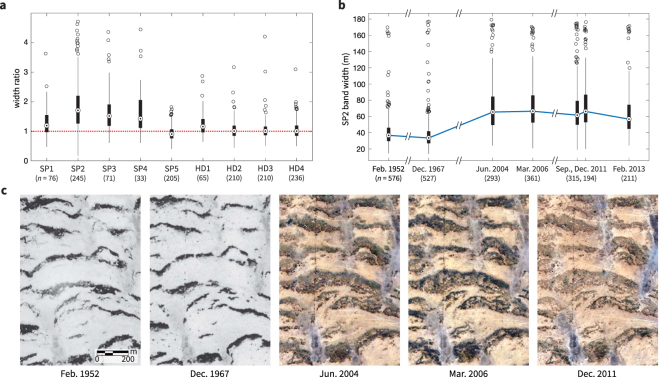
Bands widen appreciably in the direction of slope in the most heavily human-impacted areas, SP1–SP4. (**a**) The ratio of widths measured in a recent image to widths measured in a 1952 photograph are shown for all study areas. (**b**) The band widths measured in SP2 are shown at seven points in time. Widths change little between 1952 and 1967, and nearly double between 1967 and 2004. (**c**) An example of band widening in SP2 (9.73°N, 48.55°E). Images courtesy of the Bodleian Library, U.S. Geological Survey, and DigitalGlobe Foundation.

We quantified aspects of vegetation dynamics in all study areas using automated transect measurements of individual bands (Methods, Supplementary Information). Most notably, we found that bands have widened appreciably in the direction of slope in the areas SP1–SP4, while band widths remained approximately constant in the other study areas (Fig. 4, Table 1). This widening, which results in increased vegetation cover, is surprising in light of the apparent steep increases in human activity in these areas.

We computed the ratio of band widths measured in recent imagery to the widths in 1952. The median ratio among bands in each area exceeded 1.2 in SP1–SP4 (Fig. 4a). The most substantial widening occurred at SP2, where the median ratio is 1.7. We measured band widths at SP2 using additional images taken in 1967, 2004, 2006, 2011, and 2013. We found that widths did not change between 1952 and 1967, and then nearly doubled between 1967 and 2004 (Fig. 4b,c). From 2004 onward, median band width held approximately constant. Analyses over multiple time points at SP1, SP3, and SP4 showed a similar pattern (Supplementary Figure S8). Since recent images were taken in a variety of seasonal and rainfall-history conditions (Supplementary Figure S1), we conclude that the widening observed in SP1–SP4 is not an artifact of seasonality. Moreover, we do not observe widening at nearby area SP5, which strongly suggests that non-climatic factors have driven the apparent changes in SP1–SP4.

**Figure 5:**
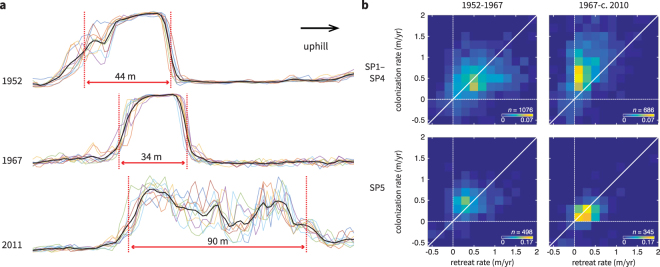
Bands widen in areas SP1–SP4 due to increased upslope edge migration (colonization) and decreased downslope edge migration (retreat). (**a**) Example image intensity profiles along a transect in SP2. Profiles along multiple parallel transects are shown in color, and the mean profile is shown in black. The estimated band edges are indicated in red. Intensity values are normalized to the same scale for comparison. (**b**) Bivariate distribution of front and back migration rates shown for the periods 1952–1967 (first column) and 1967–c. 2010 (second column), with study areas SP1–SP4 (first row) compared in aggregate with SP5 alone (second row). Band widening in SP1–SP4 results from front migration rates increasing and back migration rates decreasing during the period 1967–c. 2010.

Vegetation bands in Africa and North America are reported to migrate uphill over time due to vegetation colonization at the upslope edge of the band and mortality-driven retreat at the downslope edge^1,28^. In all our study areas, we similarly observed that bands gradually migrate uphill over six decades (Table 1). In addition, we found that bands widen at SP1–SP4 due to increased uphill migration rates at the upslope edges of the bands (colonization rate) and to decreased rates at the downslope edges (retreat rate) (Fig. 5). Between 1952 and 1967, both colonization and retreat rates are comparable in all Sool Plateau study areas, resulting in band widths that remain unchanged over this period. Between 1967 and c. 2010, colonization rates increase and retreat rates decrease in SP1–SP4, resulting in band widening. In contrast, colonization and retreat rates both decrease in SP5 by the same factor during this period, resulting in slower migration and no widening in this study area.

**Table 1:** Typical slopes, ratios of band widths between new and old imagery, and band migration rates in each study area.

Area	Slope (%) *S*	Width ratio *R*	Colonization *M*_c_ (m/yr)	Retreat *M*_r_ (m/yr)	corr(*S*, *R*) (*p*, *t*, df)	corr(*S*,*M*_*c*_) ($p$, *t*, df)	corr(*S*,*M*_*r*_) ($p$, $t$, df)
SP1	0.3–0.4	0.95–1.5	0.26–0.68	0.12–0.48	−0.21 (<0.005, 8.7, 198)	−0.19 (0.01, 7.8, 199)	0.01 (0.91, 0.0, 219)
SP2	0.1–0.3	1.3–2.2	0.57–1.2	0.20–0.55	−0.12 (<0.005, 9.5, 666)	−0.1 (0.01, 6.1, 659)	0.03 (0.43, 0.6, 629)
SP3	0.1–0.3	1.0–1.7	0.23–0.79	0.08–0.51	−0.1 (0.13, 2.4, 237)	−0.09 (0.16, 2.0, 237)	−0.05 (0.47, 0.5, 219)
SP4	0.1–0.2	1.2–1.9	0.33–0.93	0.08–0.41	−0.12 (0.14, 2.2, 152)	−0.12 (0.17, 1.9, 136)	0.00 (0.97, 0.0, 137)
SP5	0.2–0.4	0.74–1.1	0.17–0.38	0.20–0.43	−0.13 (0.01, 6.4, 346)	−0.14 (<0.005, 9.1, 435)	−0.06 (0.21, 1.6, 492)
HD1	0.4–0.6	0.93–1.4	0.22–0.55	0.07–0.39	−0.13 (0.16, 2.0, 123)	−0.32 (<0.005, 16, 141)	−0.24 (0.01, 8.1, 136)
HD2	0.3–0.5	0.85–1.2	0.25–0.47	0.21–0.46	0.12 (0.06, 4.1, 289)	−0.06 (0.31, 1.1, 324)	−0.16 (<0.005, 8.5, 314)
HD3	0.3–0.5	0.87–1.2	0.26–0.48	0.22–0.46	0.03 (0.65, 0.2, 321)	−0.14 (0.01, 7.9, 420)	−0.18 (<0.005, 13, 386)
HD4	0.4–0.5	0.85–1.2	0.19–0.41	0.15–0.39	−0.07 (0.18, 1.8, 340)	−0.14 (0.01, 16, 273)	−0.16 (0.01, 7.1, 280)

Ranges shown are from the 25th to 75th percentiles. The width ratios (*R*) and upslope colonization and retreat rates, *M*_*c*_ and *M*_*r*_ respectively, were measured between 1952 and c. 2010 (images used for analysis indicated in Supplementary Table S1). Significance of correlations was assessed using a one-tailed *t*-test corrected for spatial autocorrelation^44^, and *p* values, *t* values and degrees of freedom are given.

We found evidence that migration rates and band width ratios vary inversely with local slopes, which are typically shallow and range between 0.1–0.6% (Table 1). Colonization rates are negatively correlated with slope in a majority of study areas (*p* ≤ 0.01), including the areas in the Sool Plateau where width ratios are also negatively correlated with slope (*p* ≤ 0.01). Retreat rates are only negatively correlated with slope in the Haud study areas (*p* ≤ 0.01). Since widening in SP1–SP4 occurred due to increased colonization rates, this suggests that bands tend to widen to a greater degree on shallower slopes due to faster rates of colonization. An inverse relationship between slope and migration rate seems to contradict previous investigations of a mathematical model for vegetation banding, which indicate either a negligible^29^ or an increasing relationship^30^. Moreover, a positive relationship between migration and slope is expected in the limit of vanishing slope, since the migration rate must approach zero as the anisotropy induced by the slope vanishes. The bands in regions of study may rely critically on the slopes being above some threshold and this could explain why we do not observe positive relationships between slope and migration.

### Comparison with previous theoretical predictions

Theoretical investigations of vegetation band response to environmental pressure have focused on the characteristic spacing between bands (band wavelength) because this property is observable in remotely-sensed imagery. Idealized partial differential equation models, which account for water-biomass feedbacks and transport, predict that band wavelength should increase in response to sufficient increases in environmental pressure^19–24^. We quantified band wavelength change in all study areas using the Fourier window method developed by Penny *et al*.^31^ (Methods, Supplementary Information). In areas where bands have not completely disappeared, we found that changes in wavelength are imperceptible (Supplementary Information). In parts of the human-impacted area SP4, bands look to have degraded without an apparent change in wavelength (Supplementary Figure S6b).

Band wavelength is also predicted by models to vary with local slope, though the nature of this relationship can depend on model parameters and the history of the state^29,32^. We found a significant correlation at the 5% level between wavelength and slope in only SP3 (*r* = −0.34, *p* = 0.04) (Supplementary Table S2). The (negative) sign of this correlation agrees with empirical findings in other investigations^4,28,31^.

## Discussion

In this study, we observed two distinct environmental outcomes that depend on the level of apparent human activity. In the areas with only modest increases in activity, remarkably little about the vegetation has changed. In the areas with significant increases in activity, the vegetation has degraded substantially, with bands often disappearing entirely from the landscape. Vegetation loss is not limited to land cleared for development, since we also observed loss in areas with few human-made structures. This suggests more subtle forms of relevant human impact. Roads and dirt tracks now densely cover the landscapes of many of the study areas. This may be indicative of overgrazing or biomass harvesting, both of which are known issues in the area^33^. The roads themselves likely also affect the flow and availability of water to the vegetation bands^34^, which we observed most clearly in cases where vegetation grows within dirt tracks. Due to the faintness of the tracks in many instances, we assessed the extent of roads and dirt tracks visually. Developing automated image analysis approaches for detecting faint roads and dirt tracks would enable remote-sensing proxies for human activity that may be used to monitor the Horn of Africa and other pastoral lands.

In many study areas, we observed an increase in the widths of vegetation bands driven by altered rates of upslope and downslope edge migration. Widening occurs at Sool Plateau areas SP1–SP4, and does not occur in nearby area SP5, suggesting non-climatic causes. Since widening occurred in conjunction with apparent increases in human activity, we investigated how widening may be linked to human impacts by numerically simulating a well-studied dryland vegetation model under changes in parameters associated with a possible shift from trees to grasses^13^. Although we could not infer plant composition changes from the satellite imagery, it has been documented that *Acacia* woodcutting for charcoal production is widespread in the Sool Plateau^33^, and has likely caused a decrease in woody biomass within the vegetation bands in many areas. A shift in composition that decreases woody biomass and increases grass biomass plausibly increases the overall transpiration rate, biomass yield per unit water, dispersal rate, and mortality rate of the vegetation. We found that individually increasing transpiration, yield, and dispersal rate parameters in the model cause band widening, while increasing mortality rate had the opposite effect (Supplementary Figure S11). We also found that widening can be achieved through a simultaneous increase in transpiration, biomass yield, dispersal, and mortality rate parameters (Supplementary Figure S11b). We conclude that a shift in species composition is a viable explanation for the changes we have observed in the Sool Plateau.

Band widening in the human-impacted study areas causes local increases in vegetation cover. This is seemingly at odds with our observations of band degradation within the same areas, and also with previous studies of vegetation bands in Niger reporting diminished vegetation cover accompanying greater human activity^5,25^. Our investigation of a dryland vegetation model posits that woodcutting, a relevant impact of human activity, can induce band widening. A recent theoretical study suggests also that increased pastoral grazing pressure can cause band widening and increased vegetation cover^35^. Additional remote-sensing studies could clarify the relationship between human activity and vegetation band widening. In the present study, we analyzed only a small fraction of the 1950s aerial survey photography archive. A future study making use of randomly-sampled photographs over a larger area could better characterize the prevalence of vegetation band widening, and the frequency of its association with increased human activity. A well-resolved time series of images, e.g., from the Landsat or Sentinel satellites, could be used for a phenological analysis of vegetation that permits a spatial classification of plant functional type within bands. Such information could be used to determine how plant composition varies between bands that have widened and those that have not. Future monitoring of the region could help to determine whether band widening is ultimately a transient phenomenon, and to characterize the relationship between band widening and vegetation resilience.

We have provided new evidence suggesting that human impacts modulate the width of vegetation bands, and we argue that this vegetation property represents an underutilized window into the response of dryland vegetation to environmental pressure. In recent years, the spacing between bands (band wavelength) has been a prime focus of theoretical and empirical investigations of vegetation pattern resilience. As we and others^31^ have found, the spacing between bands in nature is often irregular, making both the notion of wavelength and its measurement imprecise. In models of vegetation patterning, it is common for a range of band wavelengths to be stable over a range of environmental conditions, making wavelength changes in model scenarios of environmental change history-dependent and discontinuous^20–22,36^. In contrast, band widths are straightforward to measure in remotely-sensed imagery, and our model investigation suggests that band widths change continuously in response to parameter variation (Supplementary Figure S11). Future theoretical and empirical investigation of this vegetation property will be important to establishing its utility to dryland monitoring in the Horn of Africa.

## Methods

### Regional information

We studied areas within the Sool Plateau and Haud pastoral regions of Somalia. Both regions are generally characterized by an arid climate (aridity index = 0.04–0.1)^37^. Due to a lack of continuous rainfall station monitoring in and around our regions of study, we assessed the historical regional climate using 20th Century Reanalysis^38^ and the CPC/Famine Early Warning System Dekadal Estimates datasets (Supplementary Figure S1). Mean annual rainfall in both regions ranges between 100–300 mm. We found no evidence that rainfall conditions have changed in either region in recent decades, and we identified a warming trend in average yearly temperature of 1–2 °C over the last half-century.

Regional soils are claylike and prone to crust formation, resulting in low permeability and surface water runoff following high-intensity rainfall^33,39^. Hemming found that soils are wetter beneath bands in the Haud, indicating greater soil permeability in vegetated areas^39^. Vegetation bands in both regions are dominated by *Andropogon kelleri* grasses^26,39^. Bands also contain a mix of trees and shrubs, most notably *Acacia bussei*. In recent decades, *Acacia bussei* has diminished in abundance in the Sool Plateau due to cutting for charcoal production^33^. Disruption of traditional grazing patterns has resulted in overgrazing in many areas of the Sool Plateau, including Dhahar (SP4)^33^.

### Data

We studied approximately 260 km^2^ of imagery within the Sool Plateau and 200 km^2^ of imagery within the Haud (Fig. 2). Study areas were chosen based on a combination of factors. In particular, we wished to include areas with different development and degradation outcomes, areas with recorded soil and floristic information based on field studies, areas in geographically distinct regions, and areas featuring well-defined banding. Study area boundaries are defined by our choice of British Royal Air Force (R.A.F.) aerial survey photography, which comprise our earliest image datasets. Aerial survey photographs were taken in 1951–52 over broad areas of present-day Somalia, and specific photographs were scanned on request by the Bodleian Library at the University of Oxford. We also studied declassified reconnaissance satellite imagery taken in 1967, and DigitalGlobe imagery for dates spanning 2004–2016. Resolution of imagery used in this study ranges between 1.4–2.4 m/pixel. Satellite images taken between 2004–2016 containing red and near-infrared channels were used to compute a Soil-adjusted Vegetation Index^40^. We manually georeferenced R.A.F. scans and the 1967 reconnaissance image using visually identified control points. We estimated georeferencing error to be approximately 1–2 pixels.

We estimated local gradient within our study areas using the Shuttle Radar Topography Mission Global 1 arc second elevation dataset^41^. Because of the noise characteristics of the dataset and the low relief of our study areas, we used a second-order finite difference operator with noise-suppressing properties to estimate gradient and slope^42^ (Supplementary Figure S2).

### Visual comparison

We assessed changes in our study areas over time through a systematic visual comparison of imagery. We developed a graphical user interface in MATLAB for comparing images (Supplementary Figure S3). For each area, we split both R.A.F. scans and recent imagery into 1 km × 1 km boxes, and evaluated qualitative features within these boxes. We evaluated the extent of roads and dirt tracks, which served as our primary proxy for human pressure (Supplementary Figure S4). We also evaluated the extent of banding and defined degradation in this context as the breakdown in regularity or disappearance of banding between the earliest and most recent images (Supplementary Figure S5).

### Automated transect measurements

We quantified band widening and migration using grayscale image intensity profiles gathered along transects drawn through the bands. We used the same transects for multiple images in the same study area. We fit a top hat function to each intensity profile to extract band width (Supplementary Figure S7). Intensity measurements were gathered along multiple parallel transects for each band, and data points with high variance in measured widths were discarded.

### Fourier analysis

We measured changes in band wavelength using the Fourier window method by Penny *et al*.^31^ The method measures band wavelength and orientation in a sliding window using a 2D FFT, and computes a uniqueness metric based on the unimodality of the radially-binned power spectrum. We discarded data points which correspond to sites without banding using a manually-drawn mask. We additionally discarded data points with uniqueness values below a threshold.

### Model simulations

We simulated the model by Klausmeier^13^, a conceptual model describing biomass-water interactions in dryland environments, in one spatial dimension. We estimated the sensitivity of band width to parameter changes. We obtained the initial parameter set from the values and ranges given in^13^. Parameters stated in^13^ to differ between grasses and trees are set at intermediate values so that the spatial scale of banding resembles those in our regions of study. The time scale of migration was similarly tuned using the downhill water flow rate parameter. We simulated the model using the exponential time differencing fourth-order Runge-Kutta pseudospectral scheme^43^.

### Statistics

We assessed the significance of correlations between quantities computed from transect and Fourier analyses using a one-tailed paired *t*-test corrected for spatially autocorrelated data^44^, implemented in SpatialPack for R^45^.

### Data availability

Data and code supporting the findings of this study are available at 10.21985/N2GQ1G.

## Electronic supplementary material

Supplementary information
