# Supplementary material for: Signatures of human impact on self-organized vegetation in the Horn of Africa
Source: Sci Rep. 2018 Feb 26;8:3622. doi: 10.1038/s41598-018-22075-5 (PMC5827523; doi:10.1038/s41598-018-22075-5)
Supplement: Supplementary file 1 — Supplementary information [file 41598_2018_22075_MOESM1_ESM.pdf]

Supplementary information

Signatures of human impact on self-organized vegetation in the Horn of Africa

Karna Gowda[1], Sarah Iams[2], and Mary Silber[*3]

[1]Department of Engineering Sciences and Applied Mathematics, Northwestern University, Evanston, IL 60208, USA
[2]Paulson School of Engineering and Applied Sciences, Harvard University, Cambridge, MA 02138, USA
[3]Committee on Computational and Applied Mathematics, and Department of Statistics, University of Chicago, Chicago, IL 60637, USA

Contents

S1 Regional information **2**
S1.1 Climate . . . 2
S1.2 Vegetation . . . 2
S1.3 Soil . . . 3

S2 Data **4**
S2.1 Imagery . . . 4
S2.2 Elevation . . . 6

S3 Visual comparison **6**
S3.1 Protocol . . . 6
S3.2 Highlighted examples . . . 8

S4 Automated transect measurements **10**
S4.1 Protocol . . . 10
S4.2 Sool Plateau measurements . . . 12

S5 Fourier analysis **12**
S5.1 Protocol . . . 12
S5.2 Wavelength change . . . 14
S5.3 Wavelength-slope correlations . . . 15

S6 Model simulation **15**

*msilber@uchicago.edu

S1 Regional information

We studied imagery in areas located within the Sool Plateau and Haud pastoral regions of Somalia. Sool Plateau study areas are located approximately 50 km west of Gardo, and Haud areas are located approximately 40 km south of Las Anod. Areas were chosen for this study based on a combination of factors; in particular, we wished to include areas with different development and degradation outcomes, areas with recorded soil and floristic information based on field studies, areas in geographically distinct regions, and areas featuring well-defined banding. Detailed information about areas and imagery is given in Section S2.1 and Table S1.

S1.1 Climate

Both Sool Plateau and Haud pastoral regions are characterized by an arid climate (aridity index = 0.04-0.1) [1]. Rainfall in Somalia is bimodally distributed between the Gu season, spanning Apr.-May, and the Deyr season, spanning Oct.-Nov. Separating the rainy seasons are two dry seasons, Xagaa (Jun.-Sept.) and Jilaal (Dec.-Mar.). Deyr rainfall events are typically shorter and less significant than those of the Gu. The Jilaal season is typically the hottest and driest time of year.

Due to a lack of continuous rainfall station monitoring in and around our regions of study, we assessed the historical regional climate using climate reanalysis and remotely-sensed rainfall estimation datasets. The 20th Century Reanalysis (V2c) dataset assimilates surface pressure observations, sea-surface temperature, and sea ice extent into a global climate model to obtain a reconstruction of Earth's climate spanning 1871-2011 [2]. The V2c dataset is available at 6-hour temporal and 2° spatial resolution. The coarse spatial resolution of the data prevents us from distinguishing the Sool Plateau and the Haud regions. Uncertainty estimates can be derived from 56 replicate model simulations. The CPC/Famine Early Warning System Dekadal Estimates (RFEv2) dataset uses satellite microwave sensing and ground station observations to estimate total rainfall over the African continent for dates spanning 2000 to present. RFEv2 data is available at daily intervals and 0.25° spatial resolution.

To assess the rainfall conditions surrounding our imagery datasets, we obtained annual total rainfall estimates from the V2c and RFEv2 datasets (Figure S1a-d). In the absence of ground confirmation, we exercise caution in interpreting the V2c estimates for the 1940s-60s, and conclude only that there is no evidence that rainfall conditions have improved in either region in recent decades. We speculate that rainfall conditions surrounding the 1952 and 1967 datasets were above average. We also speculate that conditions have either declined or reverted to a regional mean in recent decades.

We assessed rainfall conditions for the recent imagery in greater detail by calculating seasonal rainfall totals from the RFEv2 dataset (Figure S1c-d). In the Sool Plateau, the images used in this study were taken in a variety of seasons and rainfall history conditions. The 2004 image was taken shortly after the return of rains that followed a very severe multi-year drought. The 2006, 2011, and 2013 images were taken amid more typical rainfall conditions. The 2016 image was taken during a period of severe drought in northeastern Somalia, which is ongoing at the time of writing. In the Haud, the images used in this study were taken in 2012 and 2016, years with robust rainfall during the wet seasons.

We examined regional temperature history using surface temperature estimates from the V2c dataset. We computed the average yearly temperature, defined as a yearly average over the daily midpoint between minimum and maximum temperatures (Figure S1e). We identified a distinct linear warming trend between 1960 to the present of 1-2 °C.

S1.2 Vegetation

Field investigations in the 1950s-60s found vegetation bands in the Sool Plateau and the Haud to be dominated by *Andropogon kelleri* grasses [3, 4]. The bands also usually contain a mix of trees and shrubs. Long-lived *Acacia bussei* trees often populate the bands. In many of our study areas, bands occur on relative low-ground (e.g., in channels), while a more uniform cover occurs on the surrounding higher ground. Greenwood attributes this difference to the clay content of the soil: higher-ground areas have a lower clay content, and thus can support a greater density of vegetation [5].

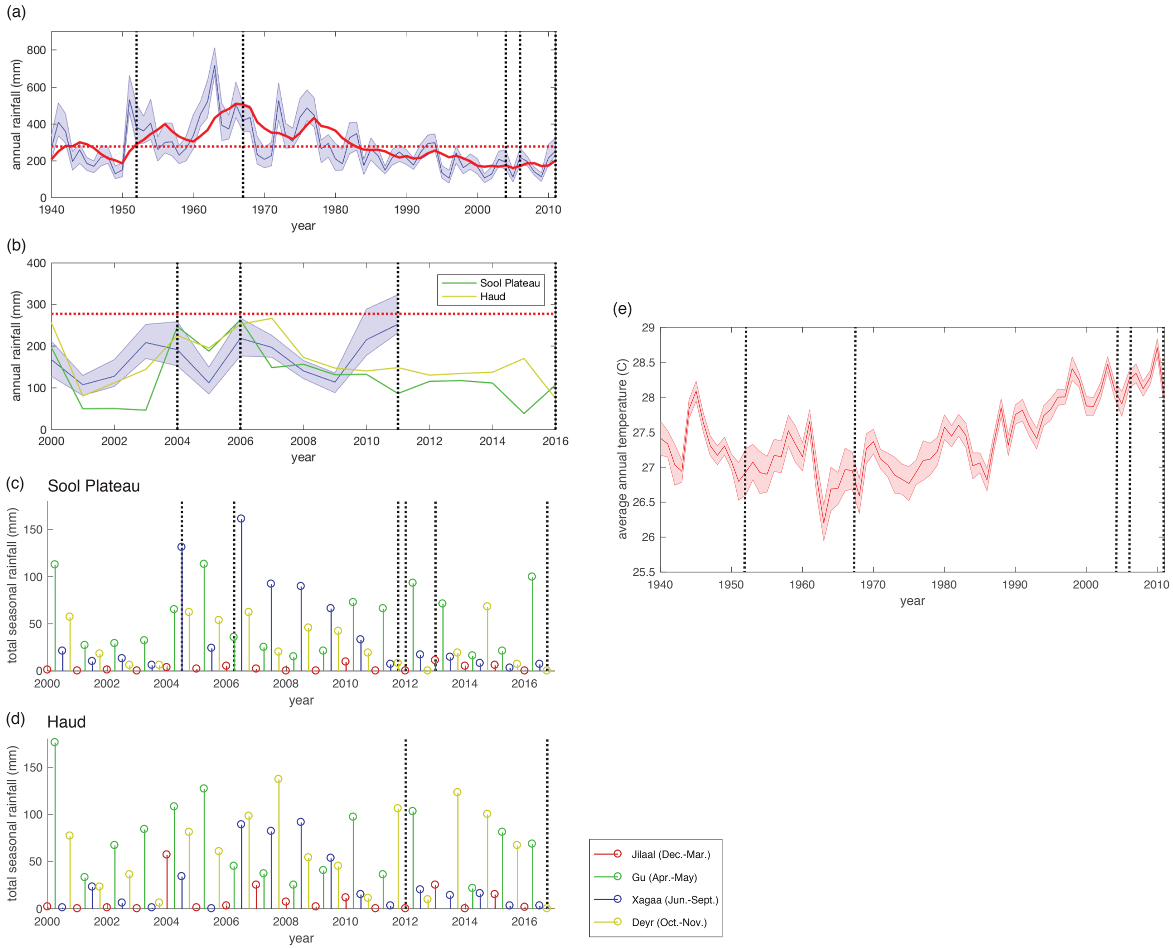

Figure S1: Annual total rainfall estimates from V2c and RFEv2 datasets, and temperature estimates from V2c dataset. (a) shows the median V2c annual total rainfall estimates between 1940 and 2011 for a large region which includes both Sool Plateau and Haud sites. The area between 25th and 75th percentiles is shaded. The running average of median precipitation over the previous 5 years is plotted with a red solid line. The average rainfall over the entire interval is indicated with a red dashed line. (b) shows V2c (2000-2011) and RFEv2 (2000-2016) annual rainfall datasets. The average V2c rainfall estimate over 1940-2011 is indicated with a red dashed line. (c) shows seasonal rainfall totals in an area containing the Sool Plateau study areas, and (d) shows totals in an area containing Haud study areas. (e) shows average yearly temperature (°C) computed from V2c reanalysis dataset. Average yearly temperature is defined as a yearly average over the daily midpoint between minimum and maximum temperatures. One standard deviation about the mean based on 56 reanalysis simulations is indicated with shading. Dates of imagery datasets are indicated in black dashed lines.

In recent decades, *Acacia bussei* has diminished in abundance in the Sool Plateau due to cutting for charcoal production, as it is considered the most lucrative species for this purpose [6]. Disruption of traditional grazing patterns has resulted in overgrazing in many areas of the Sool Plateau, including Dhahar (SP4).

S1.3 Soil

Soils studied around areas with vegetation bands in the Haud [4] and Sool Plateau [6] are claylike and finely textured. In both regions soils appear prone to crust formation and soil pore plugging, resulting in low permeability and surface water runoff following high-intensity rainfall. Hemming found that soils are wetter beneath bands in the Haud, indicating greater soil permeability in vegetated areas [4]. Soils in the Sool

Plateau thinly cover a limestone bedrock, which is exposed in some parts of our study areas.

S2 Data

S2.1 Imagery

We studied approximately 260 km^2 of imagery in areas of the Sool Plateau and 200 km^2 of imagery in areas of the Haud. Study area boundaries are defined by our choice of British Royal Air Force (R.A.F.) aerial survey photography, which comprise our earliest image datasets. Aerial survey photographs were taken in 1951-52 over broad areas of British Somaliland, and are archived at the Bodleian Library at the University of Oxford. The aerial photographs used in this study were scanned on request by the Bodleian Library using British Ordnance Survey maps to identify images.

The coordinates of study areas and additional information about imagery used in this study are given in Table S1. R.A.F. images were scanned at a nominal resolution of 1.4-2.5 m/pixel. We obtained more recent imagery through the USGS and DigitalGlobe Foundation. We purchased declassified reconnaissance satellite imagery[1] taken in 1967 from the USGS Earth Explorer site. We downloaded freely available OrbView-3 imagery taken in 2005 from the USGS Earth Explorer site. We were granted QuickBird-2, WorldView-1, and WorldView-2 imagery for dates spanning 2004-2016 by the DigitalGlobe Foundation.

We manually georeferenced aerial survey photograph scans in ArcMap 10.3 against the ArcGIS World Imagery layer using the WGS84 Web Mercator coordinate system (EPSG:3857). Because vegetation bands migrate over time, we could not match scans with geospatial coordinates using the appearance of the bands themselves. Instead we relied upon apparent geological features, such as limestone outcrops, and geometrically distinct clusters of individual trees or shrubs that persisted over time. Aerial survey photographs were matched using no fewer than 10 control points per image, and were aligned by fitting a projective transformation. Control points were stored in a tab-delimited file. A projective transformation is overdetermined for greater than 4 control points, and the root mean squared error (RMSE) of the transformed control points served as our estimate of georeferencing error.

To estimate the effect on RMSE of adding additional control points, we used a resampling procedure that calculates the alignment RMSE for different subsets of the control points. For an image that was aligned using n control points, we computed the RMSE for permutations of $5 \leq k \leq n$ control points. The average RMSE values over the permutations were then computed for each value of k. In this procedure, if the total number of such permutations $\binom{n}{k}$ exceeded 10^3, a random sampling of 10^3 distinct permutations were used. Otherwise, all permutations were used. The resulting curves were well fit by the saturating function $a\tilde{k}/(1 + b\tilde{k})$, where $\tilde{k} = k - 5$, to extrapolate the saturating value of the average RMSE curve. In all cases the saturating RMSE value was comparable to the resolution of the imagery, suggesting an alignment error on the order of 1-2 pixels.

A reconnaissance satellite image was also manually georeferenced in ArcMap 10.3 using a third-order polynomial transformation with 18 control points. The image covers a much broader area than the aerial photographs, and due to distortions arising from the imaging methodology a projective transformation did not produce a suitable fit[2]. RMSE of this alignment is 0.94 m, which is on the order one pixel. DigitalGlobe imagery was pre-georeferenced, and precise alignment with the ArcGIS World Imagery layer required only manual translation.

Most recent satellite imagery used in this study contain data sensed at different frequency channels. The red, green, and blue channels were used for visualization, and the red and near infrared channels were used for computing the Soil-adjusted Vegetation Index (SAVI) [8], an index of photosynthetic activity:

$$\text{SAVI} = \frac{NIR - R}{NIR + R + L}(1 + L).$$

NIR is the near-infrared reflectance value, and R is the red reflectance value. We computed reflectances from raw pixel intensity values using radiometric calibration adjustment factors given by DigitalGlobe[3] and

[1]Corona program, Mission No. 1102-1
[2]A third-order polynomial fit is used for comparable imagery in [7]
[3]https://www.digitalglobe.com/resources/technical-information

Area	(Lat, Lon)	Data area (km^2)	Date	Res. (m)	Channels used	Source	Sensor
SP1	(9.79°, 48.55°)	57	02/22/1952†	1.9*	Grayscale scan	Bodleian	
			12/12/1967	2.0*	Grayscale scan	USGS	KH-4B
			06/10/2004	2.4	R,G,B,NIR	DigitalGlobe	QuickBird-2
			03/23/2006	2.4	R,G,B,NIR	DigitalGlobe	QuickBird-2
			09/29/2011†	2.0	R,G,B,NIR	DigitalGlobe	WorldView-2
			12/03/2011	2.0	R,G,B,NIR	DigitalGlobe	WorldView-2
			02/24/2013	2.0	Panchromatic	DigitalGlobe	WorldView-2
SP2	(9.72°, 48.55°)	58	02/22/1952†	1.9*	Grayscale scan	Bodleian	
			12/12/1967	2.0*	Grayscale scan	USGS	KH-4B
			06/10/2004	2.4	R,G,B,NIR	DigitalGlobe	QuickBird-2
			03/23/2006	2.4	R,G,B,NIR	DigitalGlobe	QuickBird-2
			09/29/2011†	2.0	R,G,B,NIR	DigitalGlobe	WorldView-2
			12/03/2011	2.0	R,G,B,NIR	DigitalGlobe	WorldView-2
			02/24/2013	2.0	Panchromatic	DigitalGlobe	WorldView-2
SP3	(9.60°, 48.59°)	46	11/29/1952†	1.4*	Grayscale scan	Bodleian	
			12/12/1967	2.0*	Grayscale scan	USGS	KH-4B
			06/10/2004	2.4	R,G,B,NIR	DigitalGlobe	QuickBird-2
			03/23/2006	2.4	R,G,B,NIR	DigitalGlobe	QuickBird-2
			12/03/2011†	2.0	R,G,B,NIR	DigitalGlobe	WorldView-2
			02/24/2013	2.0	Panchromatic	DigitalGlobe	WorldView-2
SP4	(9.75°, 48.83°)	58	02/22/1952†	1.6*	Grayscale scan	Bodleian	
			12/12/1967	2.0*	Grayscale scan	USGS	KH-4B
			11/06/2005	1.0	Panchromatic	USGS	OrbView-3
			08/16/2016†	2.0	R,G,B,NIR	DigitalGlobe	WorldView-2
SP5	(9.36°, 48.79°)	44	02/14/1952†	1.5*	Grayscale scan	Bodleian	
			12/12/1967	2.0*	Grayscale scan	USGS	KH-4B
			08/16/2016†	2.0	R,G,B,NIR	DigitalGlobe	WorldView-2
HD1	(8.14°, 47.21°)	46	02/17/1952†	2.5*	Grayscale scan	Bodleian	
			11/24/2016†	2.0	R,G,B,NIR	DigitalGlobe	WorldView-2
HD2	(8.14°, 47.39°)	50	02/14/1952†	2.5*	Grayscale scan	Bodleian	
			12/25/2011	2.0	R,G,B,NIR	DigitalGlobe	WorldView-2
			01/21/2012†	2.0	R,G,B,NIR	DigitalGlobe	WorldView-2
HD3	(8.06°, 47.44°)	50	01/24/1952†	2.5*	Grayscale scan	Bodleian	
			12/25/2011	2.0	R,G,B,NIR	DigitalGlobe	WorldView-2
HD4	(8.09°, 47.47°)	50	01/24/1952†	2.5*	Grayscale scan	Bodleian	
			12/25/2011†	2.0	R,G,B,NIR	DigitalGlobe	WorldView-2

Table S1: Study area locations and imagery datasets used in this study. Datasets used in visual comparison, Fourier analysis, and band statistics listed in Table 1 indicated with †. Nominal resolutions of photograph scans are indicated with *. R, G, and B denote red (630-690 nm for QuickBird-2 and WorldView-2), green (520-600 nm for QuickBird-2 and 510-580 for WorldView-2), and blue (450-520 nm for QuickBird-2 and 450-510 nm for WorldView-2) channels respectively. NIR denotes the near-infrared channel (760-900 nm for QuickBird-2 and 860-1040 nm for WorldView-2).

the imagery metadata. The parameter L is used to adjust for exposed soil surface in low-vegetation cover scenarios, and is often used in place of Normalized Difference Vegetation Index (NDVI) in dryland vegetation inference. SAVI is equivalent to NDVI for $L = 0$. For visualizing SAVI values, we used a conventional parameter value of $L = 0.5$.

S2.2 Elevation

We used NASA Shuttle Radar Topography Mission Global 1 arc second (SRTMGL1) elevation data for our upslope migration assessment and comparison of pattern properties with slope. Datasets were obtained from the USGS website[4]. Datasets are packaged in 1° latitude × 1° longitude tiles, and were projected onto the WGS84 Web Mercator coordinate system (EPSG:3857) in MATLAB 2016b. This allowed for elevation data to be matched with imagery.

Though the SRTMGL1 dataset has a nominal resolution of 1 arcsecond (∼30 m/pixel near the equator), the true resolution is closer to 45-60 m/pixel due to the manner in which data was collected [9]. The data also contains speckle which is autocorrelated at a length of 1-2 pixels, and also random error, both of which together result in average vertical error of approximately 4 m in areas like the Sahara Desert [9]. To eliminate autocorrelated errors, we subsampled the data to 3 arcsecond (∼90 m/pixel) resolution.

Speckle in the SRTM data presents a challenge to gradient estimation in areas of low relief, such as our regions of study, where in banded areas vertical change can be as little as 1 m per 500 m of horizontal change. To compute gradient fields, we used a second-order accuracy finite difference stencil with noise suppressing properties [10]. As an example, a 5 × 3 noise suppressing gradient operator as defined in [10] is

$$f = \frac{1}{32h}\begin{bmatrix} -1 & -2 & 0 & 2 & 1 \\ -2 & -4 & 0 & 4 & 2 \\ -1 & -2 & 0 & 2 & 1 \end{bmatrix},$$

where h is the discretization step size. Convolving this operator with the data array produces an approximation of the partial derivative in one direction. Operators with noise suppressing properties discussed in [10] can be computed for arbitrarily large stencil size.

Using a finite difference operator allows for a straightforward propagation of independent and identically distributed normal errors in the elevation data through the calculation of gradient and slope. We estimated the magnitude of errors in the elevation data by computing the standard deviation of residuals from a median subtraction:

```
slidingmed = medfilt2(SRTM, [5 5]); %median-filtered data
sig = std(SRTM(:)-slidingmed(:)); %standard dev of residuals
```

The standard deviation of error propagated to each component of the gradient is then

```
eps = sig*sqrt(sum(f(:).^2))/h;
```

Slope is obtained from the magnitude of the elevation gradient vector, and to leading order the gradient error value is also equal to the error propagated to the slope calculation.

We tested the sensitivity of slope calculations to varying stencil size s (which yields a $(2s+1)\times(2s-1)$ operator). We note again that the truncation error of the finite difference operator is second-order for any s. Intuitively too small a stencil size will have high measurement error, and too large a stencil size will result in oversmoothing. In Figure S2 we show the 25th, 50th, and 75th percentiles of the slope values within each study area computed over an interval of s, and indicate one standard deviation of propagated error around these values. We conclude that slope values are not sensitive to stencil size when $s \geq 15$, and we use 15 (which gives an operator of size 31 × 29) for all slope and gradient calculations. We confirmed by visual inspection that this stencil size produces smooth gradient fields that match hydrological features visible in the imagery (e.g., hills and channels).

S3 Visual comparison

S3.1 Protocol

We assessed changes over time at study areas via a systematic visual comparison of imagery. Roads can be visually identified in both the aerial photographs and the satellite imagery, and their presence and qualitative appearance served as our primary proxy for inferring the extent of human pressure. Vegetation in both the aerial photos and satellite imagery contrasts sharply with the light background of bare soil, and bands are

[4]https://e4ftl01.cr.usgs.gov/SRTM/

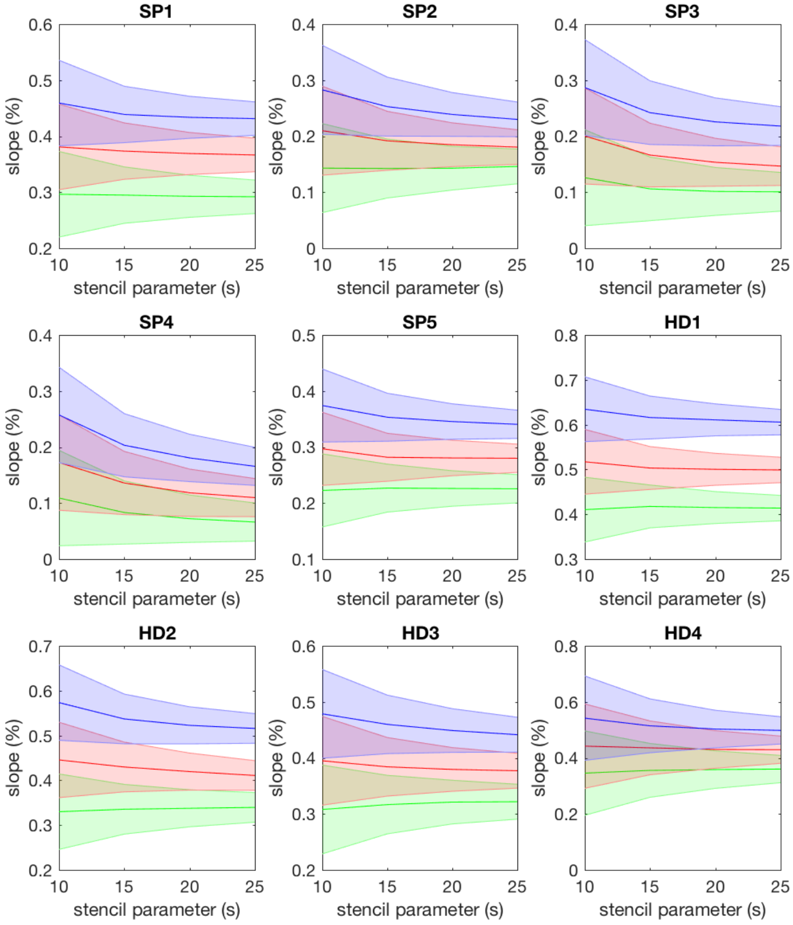

Figure S2: Sensitivity analysis of slope calculation to stencil parameter at different study areas. The 25th (green), 50th (red), and 75th (blue) percentiles of slope values are plotted as a function of the stencil parameter, and one standard deviation of propagated error are indicated in shading. Slopes are given in units of slope percentage, which is defined as 100 times the magnitude of the elevation gradient vector.

clearly identifiable. Degradation was inferred through either the breakdown in regularity or disappearance of banding.

We developed a graphical user interface (GUI) in MATLAB 2016b for visually comparing images (Figure S3). The GUI allows the user to select two imagery datasets for comparison, a georeferenced R.A.F. photograph and a more recent image. Images used for visual comparison are indicated in Table S1. The recent image is projected onto the intrinsic (row-column) coordinate system of the R.A.F. photograph, so that the data can be cleanly divided into non-overlapping 1 km $\times$ 1 km windows. The GUI simultaneously displays corresponding 1 km^2 windows of the R.A.F. photo and more recent imagery. Additionally the GUI displays a false color overlay of the two images, which was used to assess whether migration occurred. The GUI plots the local slope direction vector (computed as described in Section S2.2) on top of the overlay, which allows the user to visually assess whether the migration is in the upslope direction.

For each image window, the user is prompted to enter whether regular banding is present and whether a dense settlement is present (more than 5 structures in close proximity) using checkboxes. The user can select the extent of apparent road cover via a dropdown menu. Additionally, the user can check boxes to indicate whether band widening in the slope direction is apparent, and whether it appears that the same roads or

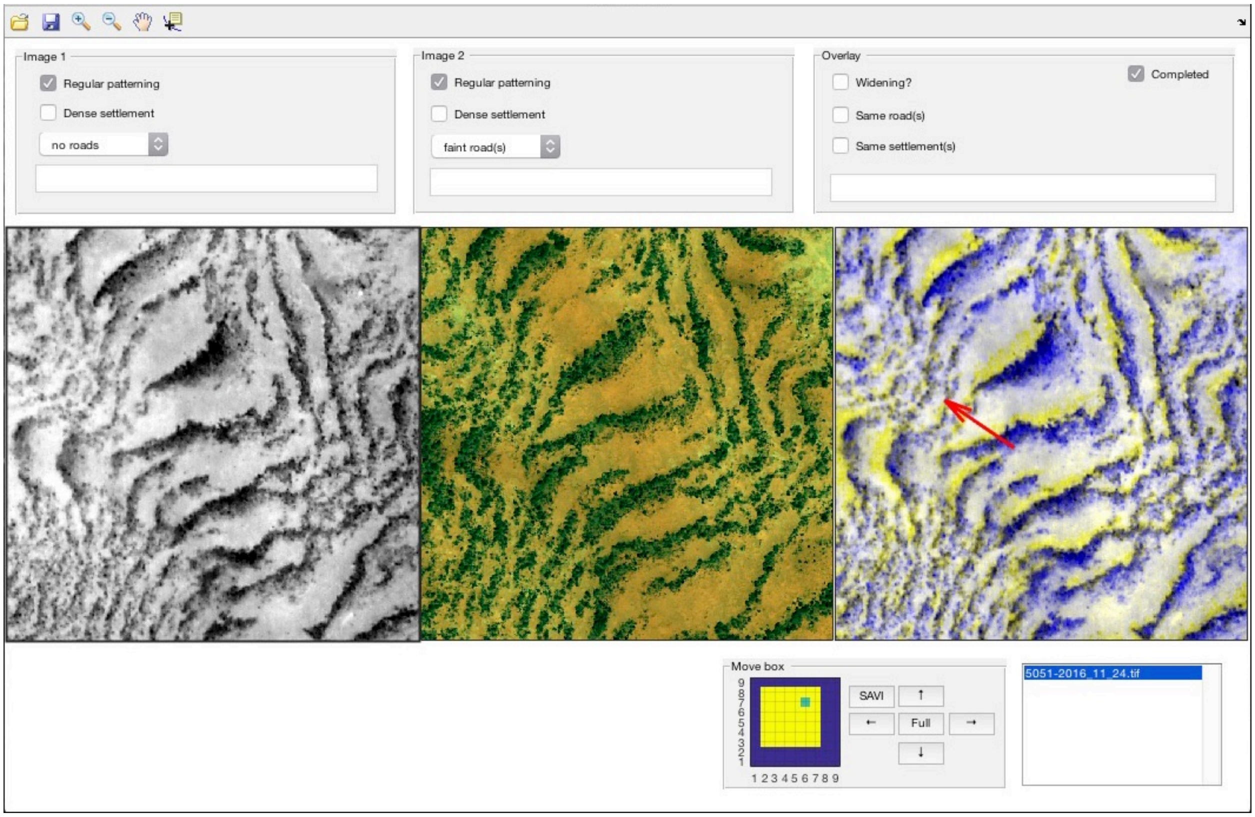

Figure S3: MATLAB GUI used for visual comparison of images. A georeferenced R.A.F. photograph and a recent satellite image are divided into corresponding windows and shown side by side (8.15° N, 47.23° E; 02/17/1952, 11/24/2016). A false color overlay of the two images is also shown, with a local slope vector overlaid to visually assess upslope migration. Blue in the false color image denotes vegetation in the R.A.F. photograph, and yellow denotes vegetation in the recent image. Images courtesy of the Bodleian Library and the DigitalGlobe Foundation.

settlements are present in both images. The user can enter comments for each image and the overlay. If the recent image contained red and near infrared channels, the Soil-adjusted Vegetation Index (SAVI) can be displayed in place of the RGB image (see discussion of SAVI in Section S2.1). The GUI selections are automatically saved to a MATLAB .mat file. When the user has finished assessing the window, the user can then navigate to different windows in the dataset using buttons.

The road cover dropdown can take on one of four states: “no roads,” “faint road(s),” “clear road(s),” and “clear, dense road(s).” The last state, “clear, dense road(s),” is taken to mean that a large number of well-incised, clearly visible roads cover a large portion of the window. Examples of these classifications are shown in Figure S4. The checkboxes are ternary; with banding, for example, a fully-checked state is taken to mean distinct banding, a half-checked state is taken to mean indistinct banding, and an unchecked state is taken to mean no banding. Examples of these classifications are shown in Figure S5.

S3.2 Highlighted examples

In many sites of the Haud study areas, we observed that human-made structures appeared to persist from 1952 to the present. In Figure S6a, we show examples of such structures.

In addition, we observed that in some areas of SP4, bands appeared to degrade without apparent change in wavelength. We show an example in Figure S6b. We verified that the bands shown have significantly lower vegetation index (SAVI) values than nearby bands in the study area, and are plausibly degraded.

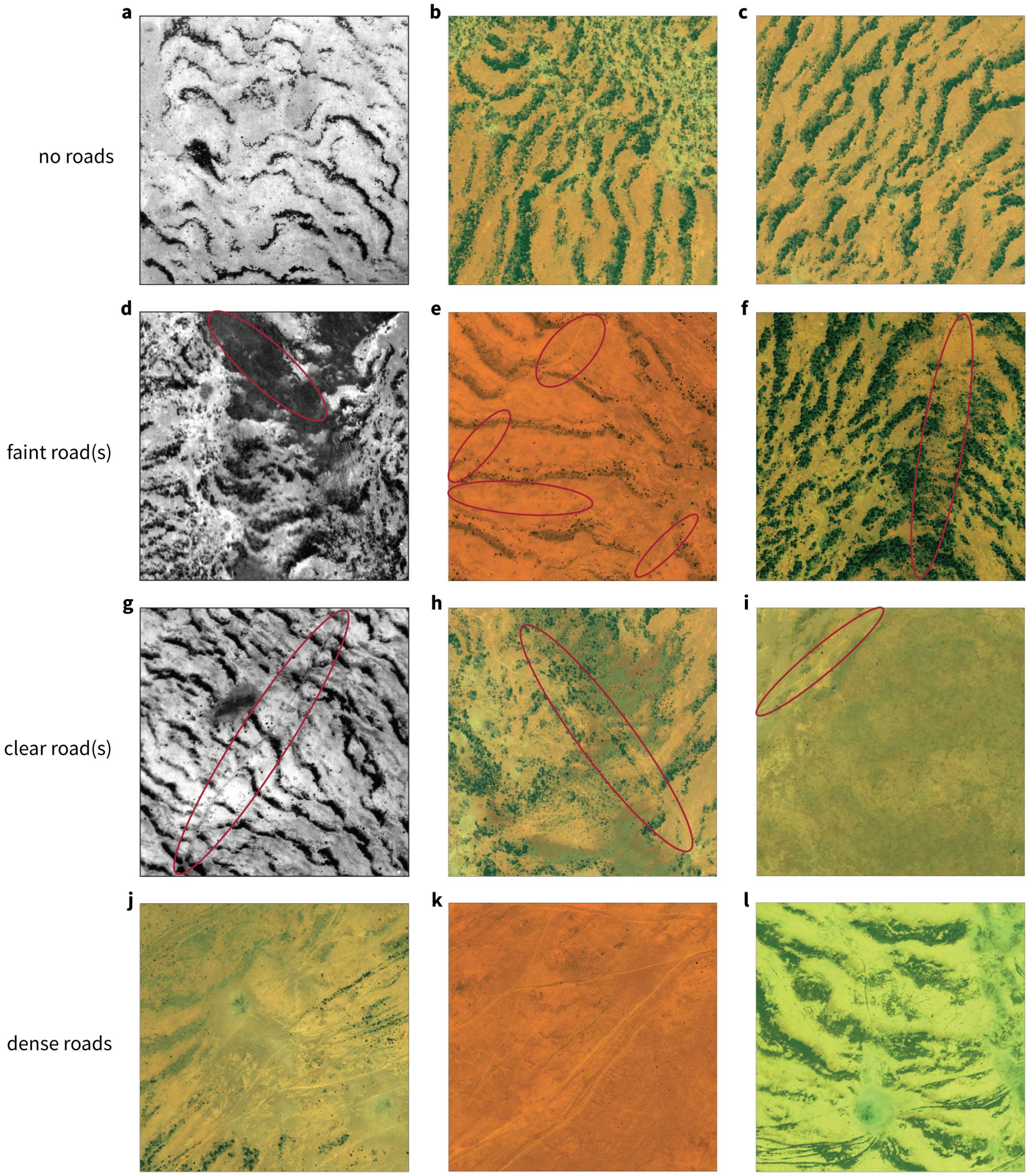

Figure S4: Examples of visual classifications of road cover state. (a)-(c) show examples of a “no roads” classification (a: SP3, 11/29/1952, grayscale; b: HD3, 12/25/2011, RGB; c: HD4, 12/25/2011, RGB). (d)-(f) show examples of a “faint road(s)” classification, where observed roads are circled (d: HD4, grayscale; e: SP5, 08/16/2016, RGB; f: HD1, 11/24/2016, RGB). (g)-(i) show examples of a “clear road(s)” classification (g: SP5, 02/14/1952, grayscale; h: HD4, 12/25/2011, RGB; i: SP3, 12/03/2011, RGB). (j)-(l) show examples of a “dense roads” classification (j: SP3, 12/03/2011, RGB; k: SP4, 08/16/2016, RGB; l: SP2, 09/29/2011, RGB). Colors shown for RGB images are normalized reflectance values computed from raw pixel intensities. Images courtesy of the Bodleian Library and the DigitalGlobe Foundation.

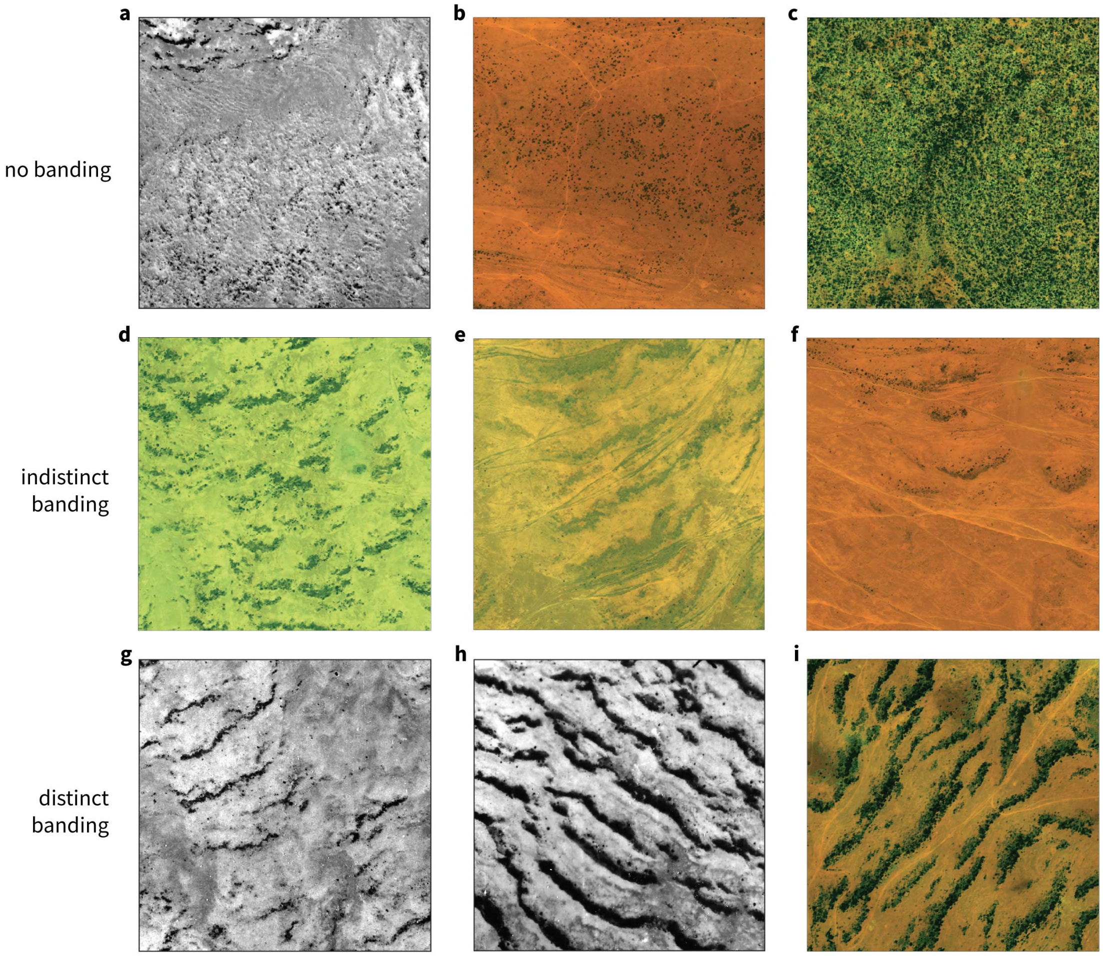

Figure S5: Examples of visual classifications of banding state. (a)-(c) show examples of a "no banding" classification (a: SP3, 11/29/1952, grayscale; b: SP4, 08/16/2016, RGB; c: HD2, 01/21/2012, RGB). (d)-(f) show examples of an "indistinct bands" classification (d: SP1, 09/29/2011, RGB; e: SP3, 12/03/2011, RGB; f: SP4, 08/16/2016, RGB). (g)-(i) show examples of a "distinct bands" classification (g: SP4, 02/22/1952, grayscale; h: SP5, 08/16/2016, grayscale; i: HD1, 11/24/2016, RGB). Colors shown for RGB images are normalized reflectance values computed from raw pixel intensities. Images courtesy of the Bodleian Library and the DigitalGlobe Foundation.

S4 Automated transect measurements

S4.1 Protocol

We quantified aspects of vegetation dynamics using automated transect measurements of individual bands. To do this, we segmented the aerial photograph to identify bands, gathered image intensity profiles along transects through the bands in direction of slope, and fit a simple top hat function to extract band width and position along the transect. We used this information to assess changes in band width over time, as well as band migration.

We eliminated the large-scale background variations in pixel intensity in the aerial photographs by subtracting a coarsely Gaussian-blurred version of the image. We then applied a manually-tuned threshold to create a binary image of the vegetation bands. We passed this binary image to the `regionprops` function in MATLAB 2016b, and extracted the areas and centroids of connected components, as well as the major and minor axis lengths and orientations of ellipses fit to the connected components. We applied manually tuned criteria on the areas and ratio between major and minor axis length to isolate the vegetation bands in the binary image.

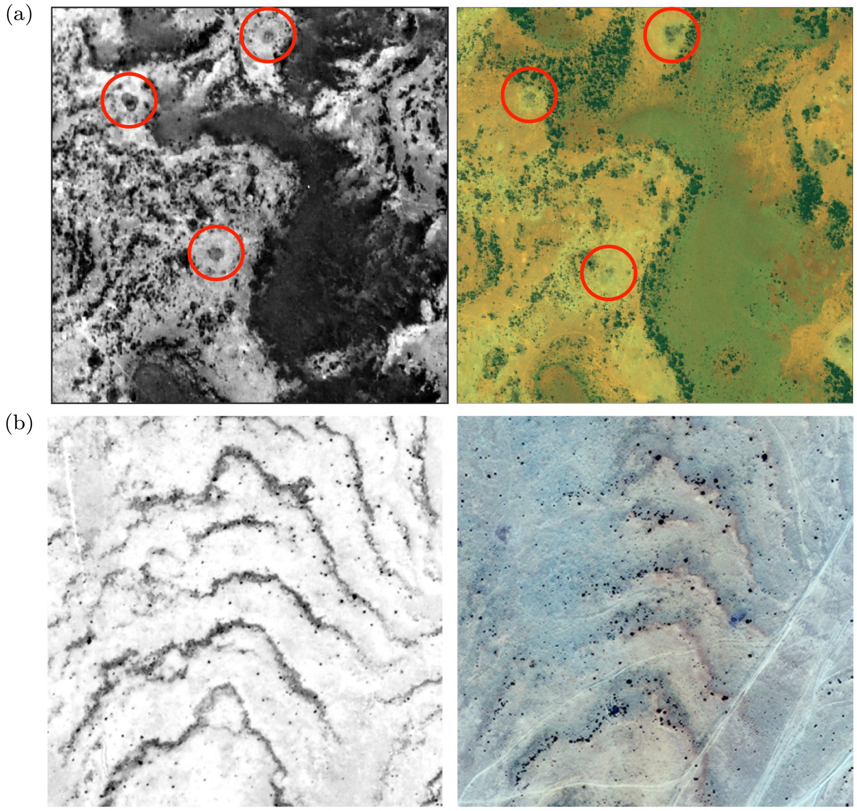

Figure S6: Highlighted examples from visual inspection. (a) shows an example at HD4 of human-made structures that appear to persist from 1952 to 2011 (8.08° N, 47.48° E; 01/24/1952, 12/25/2011). (b) shows an example at SP4 where degradation appears to have occurred without an apparent history of change in band wavelength (9.78° N, 48.84° E; 02/22/1952, 08/16/2016). Images courtesy of the Bodleian Library and the DigitalGlobe Foundation.

We then drew linear transects through the centroids of the bands in the direction of the minor axis. We visually confirmed that the minor axis direction serves as an effective proxy for the slope direction. For all study areas, transect lengths are approximately 100 pixels (∼190 m). The transects were drawn so that 25% of the transect lies downslope of the centroid, and 75% lies upslope. We did this so that the same transect could be used for both the aerial photographs and the more recent imagery, accounting for band migration upslope. In order to obtain replicate measurements for estimation of variance, we drew eight additional transects transverse to the original (four on either side). These transects are spaced approximately 4 m apart, which precludes double-sampling of pixels by adjacent transects. We then used these transects to extract pixel intensity profiles of the aerial photography and more recent imagery. We converted color images to grayscale before extracting intensities using the `rgb2gray` MATLAB function.

We fit the intensity profile with simple plateau-like curves using MATLAB's nonlinear least squares curve fitting function, `lsqcurvefit`. The curve has the form

$$f(x;\mathbf{b}) = b_1 + \frac{b_2}{2}\left[\tanh(\alpha(x-b_3)) - \tanh(\alpha(x-b_4))\right],$$

which approaches a piecewise constant function with levels b_1 and b_2 and breakpoints at b_3 and b_4 in the limit as $\alpha \to \infty$ (Figure S7). We used $\alpha = 500$. To fit this function, we rescaled all transects to lie along the interval $x \in [0, 1]$. For our data, the squared error cost function typically had many local minima, and so the result was sensitive to the initial guess for the parameters b_3 and b_4. We fit each intensity profile using 20 uniformly random initial guesses for b_3 and b_4 (such that $b_3 < b_4$), and chose among these the result with the minimum squared error. We then used the value $w = b_4 - b_3$ as the measured width of the band along the particular transect, and compared b_3 and b_4 values along a transect at different time points to measure migration.

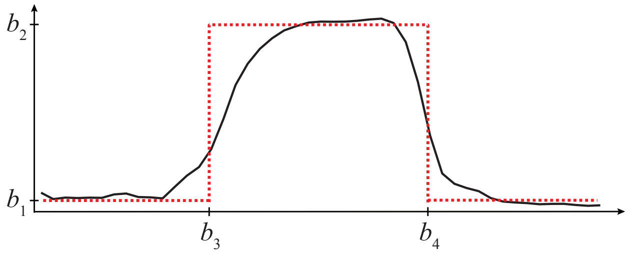

Figure S7: Schematic example of pixel intensity profile along a transect (black) and plateau function fit to the profile (red). Levels b_1 and b_2, as well as breakpoints b_3 and b_4 are indicated.

Since each band was measured using multiple parallel transects, we obtained multiple measurements of w, b_3, and b_4 for each band. We used a threshold on the standard deviation ($\sigma \leq 0.2$) of the w measurements to exclude data points where bands may have substantially degraded or disappeared, or where the measurement is likely poor for some other reason. After applying the threshold, we calculated the mean w for each remaining band at each time point.

S4.2 Sool Plateau measurements

We observed appreciable increases in band width in SP1-SP4. We measured widths at multiple time points in these areas to assess when the widening may have occurred and whether it is a seasonal phenomenon. In Figure S8, we plot the distribution of band widths in the Sool Plateau sites SP1-SP5 over time. We have reconnaissance imagery taken in 1967 for all these sites. We observe that band widths changed little between 1952 and 1967. In SP1–SP4, widths are then larger in the recent imagery (onward from 2004), and do not return to their 1952/1967 widths. In SP5, widths remain unchanged between 1967 and 2016. We conclude that band widths increased in SP1–SP4 sometime between 1967 and 2004, and that this widening is not a seasonal effect.

S5 Fourier analysis

S5.1 Protocol

We quantitatively assessed changes in band wavelength using a modification of the Fourier window method by Penny *et al.* [11]. Penny *et al.* developed the method to compute spatial maps of local wavelength and orientation from imagery over banded areas in Fort Stockton, Texas, USA. In a manner analogous to a short-time Fourier transform, the method measures wavelength and orientation in a sliding window using a 2D FFT. Vegetation banding typically contains sufficient irregularity to complicate the inference of dominant wavelength and orientation from a 2D power spectrum. The Fourier window method addresses this issue by binning power, radially for estimating wavelength and angularly for orientation, and by computing a weighted average among the contiguous bins with largest power. The method computes a uniqueness metric for both wavelength and orientation based on the distance between the maximal peak and the nearest peak with 75% of the maximal power, if present. The metric equals one if the maximal peak is the only powerful peak present, and approaches 0 as distance to the nearest powerful peak increases. In order to exclude short-wavelength noise and long wavelengths which are under-sampled for the given window size, the bins are only computed for a specified minimum and maximum wavelength interval. As presented in [11], the pattern irregularity issue is also addressed by averaging measurements over overlapping windows. We do not perform the latter step for the analyses in this study.

Penny *et al.* [11] provide MATLAB code for their method, which we modify for our analyses. We modify the main routine to take as input two images that have been resized to the same dimensions. Computations are then performed on square windows. To reduce aperiodicity effects, we apply a 2D Hamming filter (a bell-shaped function that decays to zero away from the center) to each window. We note that this filter

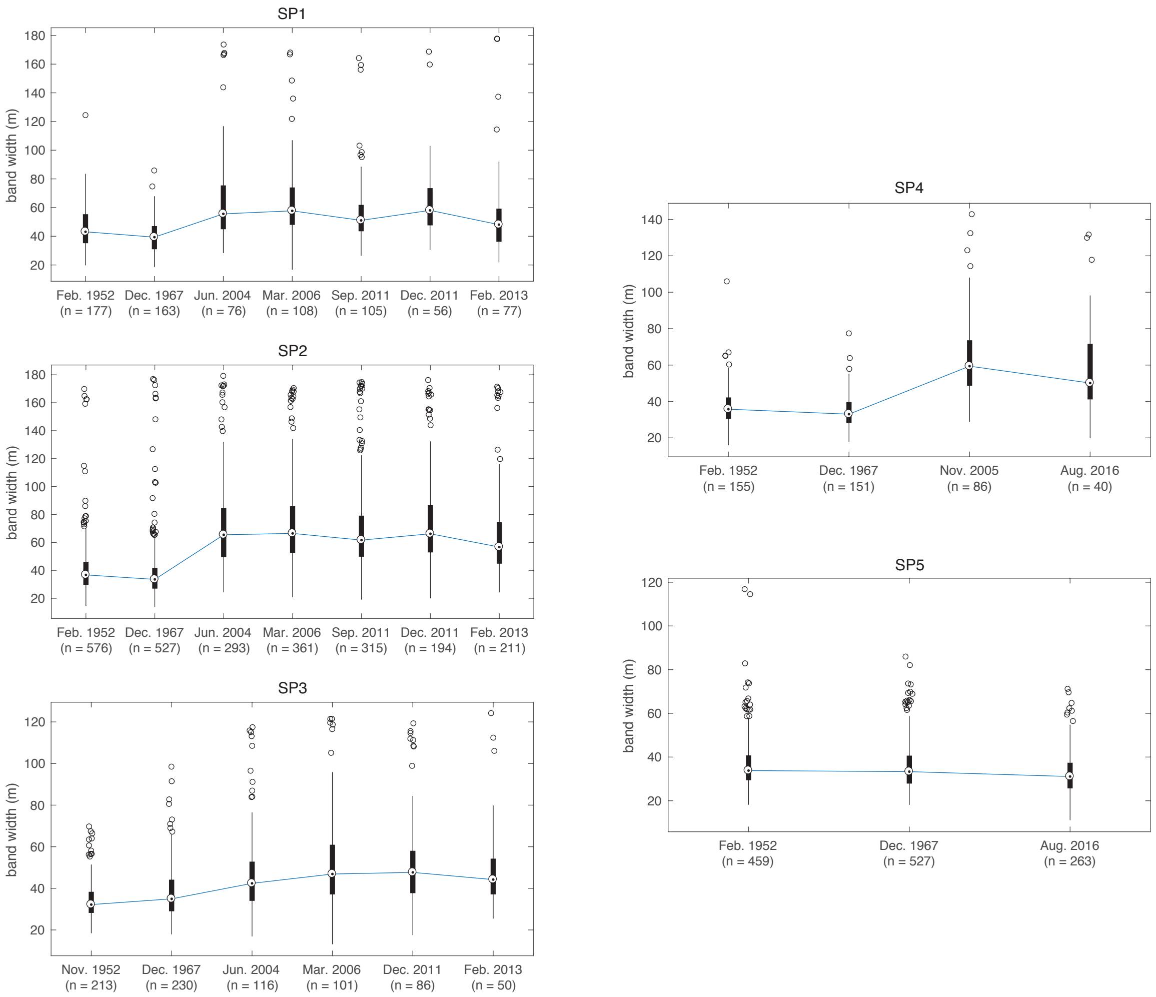

Figure S8: Band widths measured at SP1–SP5 are shown at multiple points in time. At SP1–SP4, widths change little between 1952 and 1967, and increase between 1967 and recent imagery. Widths remain approximately constant at SP5 from 1952-2016.

also has the effect of giving more weight to the central area of the image, focusing analysis on this area. Wavelengths and orientations are then computed using Penny *et al.*'s routine.

We applied this methodology to all study areas using the image pairs indicated in Table S1. In order to perform the windowing on a rectangularly-oriented dataset, we transformed the recent imagery onto the intrinsic coordinate system of the aerial photograph. We downscaled image pairs to a resolution of approximately 2.5 m/pixel to reduce computation time. We then applied two layers of preprocessing to emphasize the vegetation bands and de-emphasize other features in the imagery: we subtracted a coarsely gaussian-blurred version of the image to eliminate large scale variations in pixel intensity (such as darkening near the borders of the aerial photographs), and we applied a manually-tuned threshold to create a binary image of the vegetation bands.

For each binary image pair, we computed wavelength maps for the three square window sizes: 384 pixels (∼1 km), 512 pixels (∼1.3 km), and 768 pixels (∼2 km). After applying a Hamming filter, about 4-8 vegetation bands can be sampled in the central area of a 512 pixel window (3-5 bands for a 384 pixel window, or 6-10 for a 768 pixel window). For the binning procedure, we set the minimum wavelength to 10 pixels (∼25 m), and the maximum wavelength to one-fourth of the window size (∼240 m for a 384 pixel window, ∼320 m for a 512 pixel window, and ∼480 m for a 768 pixel window). In order to balance computation time

and even-sampling of the data, we set the step length of the sliding window to be one-fourth of the window size, resulting in adjacent windows that overlap in 75% of their area.

After wavelength maps were computed for an imagery pair, we transformed the measurements from units of pixels to units of meters. We then manually drew a mask on the imagery and applied it to the measurements in order to exclude measurements from areas without vegetation bands. Additionally we excluded measurements with wavelength uniqueness metrics smaller than 0.75. We found that in some areas, a window size of 384 pixels was too small to detect the largest wavelengths. The results of 512 and 768 pixel windows did not differ strongly, so we used the 512 pixel window computation for the results reported in the Table S2.

Area	Slope (%) S	Wavelength (m) W_1	W_2	WL change $W_2/W_1 - 1$	Slope-WL corr. corr(S, W_1) (p, t, df)
SP1	0.3–0.4	130–170	130–190	0–10%	-0.15 (0.29, 1.3, 51)
SP2	0.1–0.3	130–170	140–170	0–10%	0.02 (0.89, 0.0, 83)
SP3	0.1–0.3	120–150	140–180	0–20%	-0.34 (0.04, 4.5, 35)
SP4	0.1–0.2	130–160	150–180	0–20%	0.08 (0.53, 0.4, 67)
SP5	0.2–0.4	120–140	120–140	0–10%	-0.23 (0.06, 3.7, 63)
HD1	0.4–0.6	80–100	80–120	0–10%	-0.25 (0.33, 1.0, 16)
HD2	0.3–0.5	90–110	90–120	0–10%	-0.19 (0.20, 1.7, 45)
HD3	0.3–0.5	80–100	80–110	0–10%	-0.14 (0.17, 1.9, 94)
HD4	0.4–0.5	100–120	100-120	0–10%	0.08 (0.58, 0.3, 47)

Table S2: Band properties measured using a modification of the Fourier window method by Penny *et al.* [11]. Ranges shown are the 25th and 75th percentiles. Wavelengths W_1 were measured in the R.A.F. aerial photography datasets, and W_2 were measured in recent satellite imagery datasets. Slopes were computed from the SRTM 1 arc-second elevation dataset. Significant negative correlation highlighted red. Significance of correlations was assessed using a t-test corrected for spatial autocorrelation [12], and p values, t values and degrees of freedom are given in parentheses.

S5.2 Wavelength change

Given spatial maps of wavelength for a pair of images, we computed change maps where elements are given by

$$W_2^{i,j}/W_1^{i,j} - 1, \tag{1}$$

where $W_1^{i,j}$ is the wavelength in the first image at position (i, j), and $W_2^{i,j}$ is the wavelength in the second image at (i, j) (Figure S9). Typical ranges of the computed changes at each study area are given in Table S2. Note that for computing change maps, we have manually masked out areas with no banding, and we have also excluded measurements with a wavelength uniqueness metric smaller than 0.75. We made the latter choice to reduce the incidence of falsely detected changes between the maps, reasoning that measurements in areas with multiple dominant band wavelengths are error prone. Typical change ranges between 0–10% for all study areas except SP3 and SP4, where change ranges between 0–20%.

Our previous visual inspection suggested that there were no obvious systematic changes in wavelength at any study area, except perhaps for those associated with isolated instances of band loss in human-impacted areas. We visually reinspected areas where measured change was greater than 25% in magnitude. In some cases, it appears that these detected changes occur due to the loss of an individual band, often near evidence of human activity (Figure S10). In Figure S10b-e, significant road cover appears in the interband areas of the recent imagery, and are likely related to the loss of bands in these areas. In most cases, however, we saw no clear indication for the detected wavelength changes, and attributed these false detections to wavelength measurement error that arises due to the irregularity of the banding.

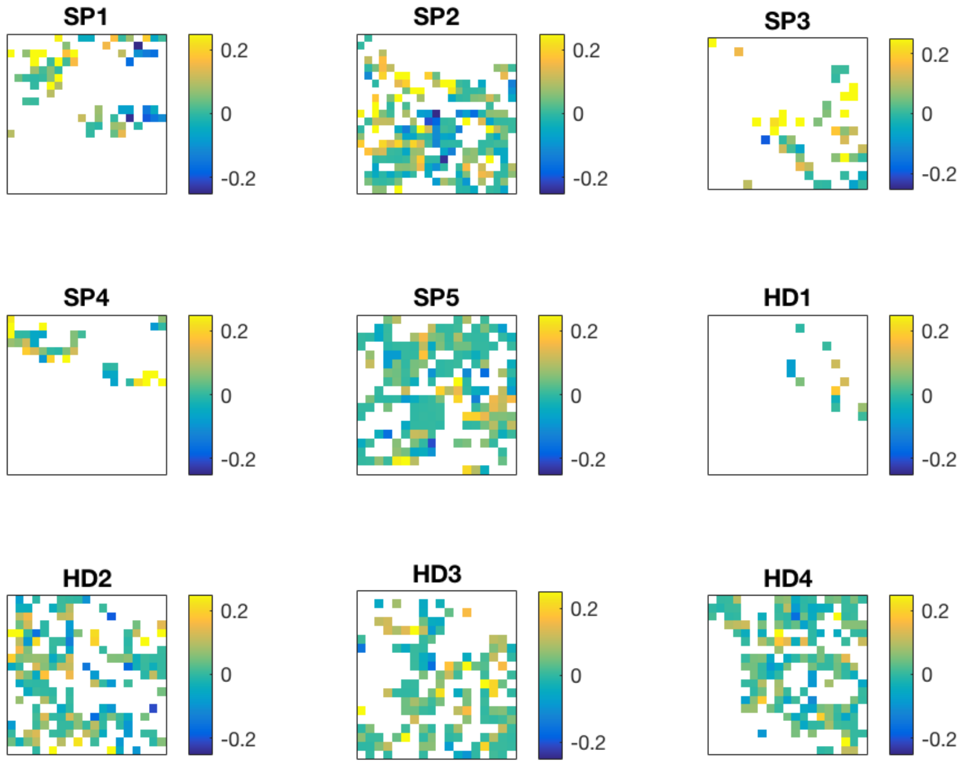

Figure S9: Wavelength change maps for all study areas. Wavelength change defined in (1), and computed between image pairs indicated in Table S1. White pixels indicate data points in areas without banding or areas with sufficiently low values of the computed wavelength uniqueness metric. All color axes are scaled to the interval [-0.25,0.25].

S5.3 Wavelength-slope correlations

We computed the correlation between local wavelength and slope, and the results are reported in Table S2). We used slope values that are closest to the center point of the window corresponding to the wavelength/migration measurement. To assess the significance of correlations, we used a paired t-test for which sample size is corrected to account for spatial autocorrelation in the data [12]. The test is implemented in the library SpatialPack for R [13]. The correlation between wavelength and slope has been empirically investigated in [11] and [7]. We found no significant correlation between slope and wavelength for our study areas except for SP3 ($r = -0.34, p = 0.04$).

S6 Model simulation

To explore how bands widen in response to parameter variation in a conceptual partial differential equation vegetation model, we simulated the model by Klausmeier [14] in one spatial dimension:

$$\begin{aligned} N_T &= -MN + JRWN^2 + D_N N_{XX}, \\ W_T &= A - LW - RWN^2 + VW_X. \end{aligned} \tag{K99}$$

Descriptions, units, and values of the parameters used are given in Table S3. The parameter set used for K99 is based on the values given in [14]. Parameters which are stated in [14] to differ between grasses and trees (M, J, and R) are set at intermediate values so that the spatial scale of banding resembles the scales in our regions of study. Water flow rate V was also approximately tuned so that a comparable time

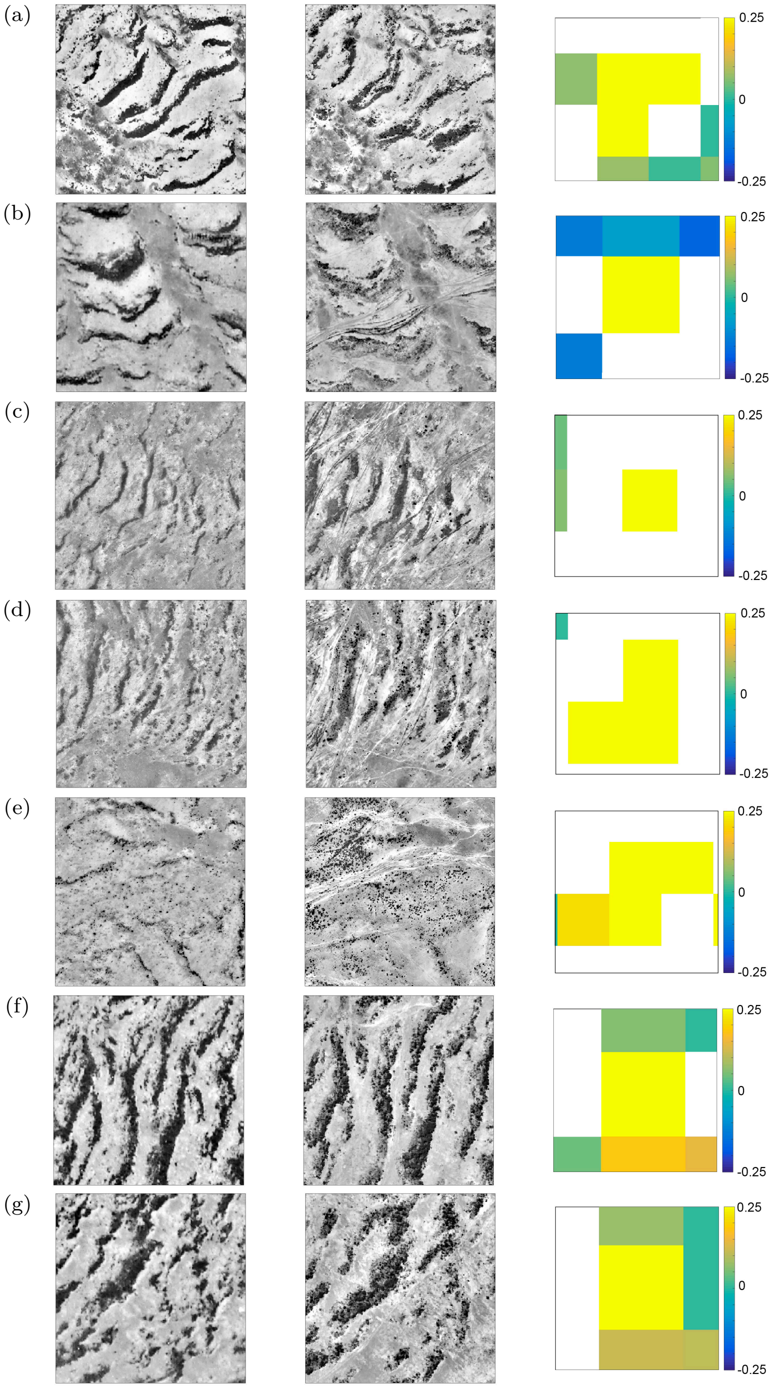

Figure S10: Areas where detected wavelength change corresponds to band loss or degradation. The first column is R.A.F. aerial photography, the second column is recent satellite imagery, and the third column is the detected wavelength change map. Locations of examples are as follows: (a) SP1 (9.77° N, 48.57° E; 02/22/1952, 09/29/2011), (b) SP2 (9.72° N, 48.54° E; 02/22/1952, 09/29/2011), (c) SP3 (9.60° N, 48.60° E; 11/29/1952, 12/03/2011), (d) SP3 (9.61° N, 48.61° E; 11/29/1952, 12/03/2011), (e) SP4 (9.76° N, 48.85° E; 02/22/1952, 08/16/2016), (f) HD4 (8.07° N, 47.46° E; 01/24/1952, 12/25/2011), and (g) HD4 (8.12° N, 47.46° E; 01/24/1952, 12/25/2011). Images courtesy of the Bodleian Library and the DigitalGlobe Foundation.

Parameter/variable	Units	Description	Value
A	mm H_2O yr^{-1}	mean annual rainfall	150
L	yr^{-1}	evaporation rate	4
J	kg m^{-2} (mm H_2O)$^{-1}$	biomass yield per unit H_2O	0.0025
M	yr^{-1}	mortality rate	0.75
R	mm H_2O yr^{-1} (kg dry mass)$^{-2}$	transpiration rate	50
D_N	m^2 yr^{-1}	plant dispersal rate	1
V	m yr^{-1}	water flow speed	35
N	kg dry mass m^{-2}	plant biomass	
W	mm H_2O (or kg H_2O m$^-2$)	water	
X	m	spatial dimension along	
T	yr	time	

Table S3: Parameters and variables for the model by Klausmeier [14].

scale of migration is reproduced in the simulations to that of our regions of study. We set the mean annual rainfall parameter to 150 mm, which is within the range of typical rainfall levels in our regions of study. We simulated K99 using the ETDRK4 explicit pseudospectral scheme [15], with 2048 grid points, a 1000 m domain, and a time step of 0.01 years.

We performed a sensitivity analysis to estimate the linear response of band width and peak band biomass to changes in the parameter set. We began each simulation using the parameter set shown in Table S3 with an initial state of the uniform equilibrium value plus small-magnitude spatial noise. We evolved the initial state to 10,000 years to obtain an equilibrium migrating patterned state. We then began a set of perturbation simulations, where in each we perturb one value in the parameter set listed in Table S3 by a percentage between 5 and 100% that is manually tuned to produce a 5-10% response in width ratio. We then evolve the initial equilibrium patterned state by 50 years. The resulting patterned states are pulselike (Figure S11a), and we measured widths by thresholding using a small value (10^{-3}). We show the width ratios for all parameter perturbation simulations in Figure S11b, where the ratios are computed by dividing band widths in the perturbed simulations by the widths from the initial patterned state. Increasing A, J, R, D_N, and V and decreasing L, and M results in band width increases.

To simulate a scenario where vegetation species composition shifts from woody to grass biomass, we simultaneously increase J, R, and M by 10% and D_N by 50% (Figure S11b). Although increasing mortality by itself reduces the band width, the simultaneous increase of these four parameters results in band width increase.

References

[1] Muchiri, P. W. Climate of Somalia. Tech. Rep. W-01, FAO-SWALIM, Nairobi, Kenya (2007).

[2] Compo, G. P. *et al.* The Twentieth Century Reanalysis Project. *Quarterly Journal of the Royal Meteorological Society* **137**, 1–28 (2011).

[3] Macfadyen, W. A. Soil and Vegetation in British Somaliland. *Nature* **165**, 121–121 (1950).

[4] Hemming, C. F. Vegetation Arcs in Somaliland. *The Journal of Ecology* **53**, 57 (1965).

[5] Greenwood, J. E. G. W. The Development of Vegetation Patterns in Somaliland Protectorate. *The Geographical Journal* **123**, 465 (1957).

[6] Oduori, S. M., Alim, M. S. & Gomes, N. Environmental study of degradation in the Sool Plateau and Gebi Valley: Sanaag Region of Northern Somalia. Tech. Rep., Horn Relief and Oxfam, Netherlands (2003).

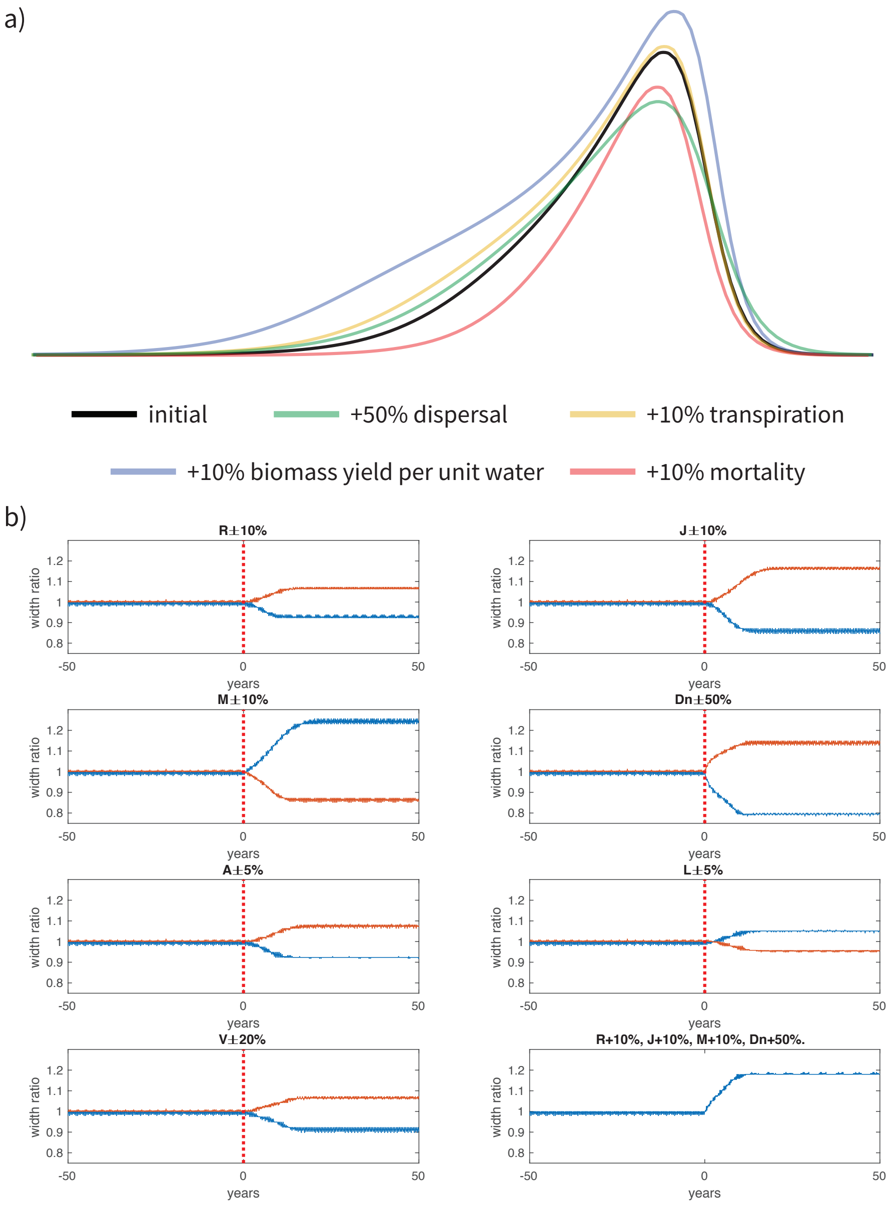

Figure S11: K99 sensitivity analysis to width ratio. (a) A comparison of equilibrium band profiles, simulated first using an initial parameter set, and then simulated after applying perturbations to individual parameters. (b) Parameters are perturbed individually by + (red) or - (blue) the percentage of the parameter indicated.

[7] Deblauwe, V., Couteron, P., Bogaert, J. & Barbier, N. Determinants and dynamics of banded vegetation pattern migration in arid climates. *Ecological Monographs* **82**, 3–21 (2012).

[8] Huete, A. R. A soil-adjusted vegetation index (SAVI). *Remote Sensing of Environment* **25**, 295–309 (1988).

[9] Farr, T. G. *et al.* The Shuttle Radar Topography Mission. *Reviews of Geophysics* **45**, 1485–33 (2007).

[10] Holoborodko, P. Noise robust gradient operators. `http://www.holoborodko.com/pavel/image-processing/edge-detection/` (2009).

[11] Penny, G. G., Daniels, K. E. & Thompson, S. E. Local properties of patterned vegetation: quantifying

endogenous and exogenous effects. *Philosophical Transactions of the Royal Society A: Mathematical, Physical and Engineering Sciences* **371**, 20120359–20120359 (2013).

[12] Dutilleul, P., Clifford, P., Richardson, S. & Hemon, D. Modifying the t Test for Assessing the Correlation Between Two Spatial Processes. *Biometrics* **49**, 305 (1993).

[13] Osorio, F., Vallejos, R. & Cuevas, F. SpatialPack: Computing the Association Between Two Spatial Processes. *arXiv.org* (2016). 1611.05289v1.

[14] Klausmeier, C. A. Regular and Irregular Patterns in Semiarid Vegetation. *Science* **284**, 1826–1828 (1999).

[15] Kassam, A.-K. & Trefethen, L. N. Fourth-Order Time-Stepping for Stiff PDEs. *SIAM Journal on Scientific Computing* **26**, 1214–1233 (2005).
